# Finding logic models for sustainable marine development that deliver on social equity

**DOI:** 10.1371/journal.pbio.3001841

**Published:** 2022-10-17

**Authors:** Yoshitaka Ota, Gerald G. Singh, Timothy Clark, Marleen S. Schutter, Wilf Swartz, Andrés M. Cisneros-Montemayor

**Affiliations:** 1 Ocean Nexus, School of Marine and Environmental Affairs, University of Washington, Seattle, Washington, United States of America; 2 Ocean Nexus, School of Environmental Studies, University of Victoria, Victoria, British Columbia, Canada; 3 Ocean Nexus, Department of Sociology, Catawba College, Salisbury, North Carolina, United States of America; 4 Ocean Nexus, Worldfish, Penang, Malaysia; 5 Ocean Nexus, Marine Affairs Program, Dalhousie University, Halifax, Nova Scotia, Canada; 6 Ocean Nexus, Resource and Environmental Management, Simon Fraser University, Burnaby, British Columbia, Canada; Smithsonian Institution, UNITED STATES

## Abstract

Sustainable development is often represented as contributing to desirable outcomes across economic, environmental, and social goals, yet policies and interventions attempting to deliver sustainable development often disagree on the order in which these categories of goals should be addressed. In this Essay, we identify and review 5 approaches (called logic models) for sustainable development in ocean systems based on existing policies and interventions and consider the evidence for their contributions to equity—the ultimate goal of sustainable development according to the UN Sustainable Development Goals (SDGs). Two of the 5 logic models prioritize economic growth and lead to social and environmental benefits, 2 prioritize environmental health as a prerequisite for sustainable economic and social benefits, and the final logic model is community driven and prioritizes social dimensions. Looking towards the 2030 maturation of the SDGs, we will need to understand what models are best suited to deliver on equity gains and prevent future inequities in development and how best to operationalize them.

## Abstract 2

Pursuing sustainable ocean development is not controversial, but the approaches towards it often are. This Essay provides an initial assessment of competing models to achieve sustainable development, and their ability to deliver on the promise of “leaving no one behind”.

## Introduction

Environmental, social, and economic dimensions of the world are interconnected and affected by the political and development policies that are set by organizations from subnational to international levels [1,2]. In particular, the social equity aspect of economic and political decisions can have considerable consequences across these social–ecological dimensions (Box 1) and, in turn, effects across these social–ecological dimensions have important equity consequences [1,3–5]. For example, the unequal distribution of benefits acquired through natural resource use, as well as the unequal distribution of the risks of environmental change, are reflections of global political and economic power [6–8]. At local scales, environmental justice groups have formed in response to pollution, habitat destruction, and development disasters that disproportionately affect minority communities and other marginalized populations owing to historic and contemporary discriminatory settlement and development policy [6]. Navigating these complex, large scale, high stakes, and interconnected dynamics towards a more desirable and equitable world is the focus of sustainable development [9,10]. How to successfully navigate these dynamics remains elusive, partly because the underlying planning models that detail the sequence of dynamics that enable sustainable development outcomes (which we refer to as logic models) and that inform development initiatives towards sustainable development objectives have largely been underexamined [10–12].

**Box 1. Glossary**

**Aggregate**

Measures and considerations of the population (or subject) as a whole. Aggregate considerations are uninterested in disparities and inequalities, but rather only track the accumulated measure. For example, gross domestic product (GDP) is a measure of the total economic activity within a country’s borders, but does not track differences in wealth among groups of people.

**Disaggregated**

Measures and considerations that evaluate how different groups fare. The focus is not on the accumulated performance.

**Ecological**

Conditions of natural systems (e.g., terrestrial, freshwater, oceanographic), biophysical flows and processes (e.g., carbon/nitrogen/phosphorous cycles, atmospheric processes), and biodiversity.

**Economic**

Aggregate economic growth and financial and infrastructural components that promote aggregate economic growth.

**Market economy**

An economic system in which the decisions on how factors of production (capitals, labor, and resources) are utilized, as well as how resulting goods and services are distributed for consumption are guided by the market.

**Price mechanism**

A system of price determination in which, in a competitive market, the unit price is set at an economic equilibrium where supply and demand intersect.

**Social**

Institutions, communities, cultural, demographic, and equity considerations (especially targeted at disaggregated levels).

**Social–ecological dimensions**

The dynamics and relationships between humans and the biophysical environment. Ecosystem services, as biophysical processes that benefit people, are an example of social-ecological dimensions.

**Use value**

The tangible features of a good (as opposed to the intangible values, such as psychological, cultural, and emotional benefits from the existence or enjoyment of a good).

Sustainable development has always been equity focused and considerate of how material well-being for people across generations requires a healthy natural environment and stable social and economic conditions [10,13]. Perhaps the most famous definition of sustainable development was posed as “development that meets the needs of the present without compromising the ability of future generations to meet their own needs,” which led to a focus on natural resource sustainability for intergenerational equity [14,15]. The modern definition, as set out in the UN Sustainable Development Goals (SDGs), is captured in the central vision of “leave no one behind,” which considers historic and contemporary equity concerns to eliminate discrimination and exclusions and promote the well-being of the worst-off, while protecting opportunities for future generations [9,15]. Although the SDGs are targets to be realized by 2030, there is continuous debate about how to reach them and how to continuously improve beyond 2030 [10,13,15,16].

Oceans and coastal systems provide an important and complex arena for understanding and undertaking sustainable development initiatives. Oceans and coastal systems are home to a large proportion of the world’s population, provide sites of economic and social–cultural importance to coastal communities, and pose difficult transboundary and multiple-use decisions for policymakers [17–19]. Moreover, ocean and coastal systems are argued to be the most directly relevant systems across all SDGs and are known to face highly consequential climate impacts with repercussions to all SDGs [3,12,17,20]. We are also currently in the UN Decade of Ocean Science for Sustainable Development, which provides an international platform for promoting research in support of sustainable development [12]. However, despite the importance of oceans and coastal systems, successfully operationalizing the sustainability and equity aspirations of sustainable development in ocean contexts remains elusive and continuously debated [12].

Though there are broad efforts and proposals to address equity and sustainability (such as the 6 transformations proposed for the SDGs or the 5 “levers” to achieve transformation by the Intergovernmental Science-Policy Platform on Biodiversity and Ecosystem Services (IPBES)) [21,22], such proposals have been criticized for their lack of procedural perspective (e.g., what programs and outcomes are prerequisites or preclusions of others) [11,12]. Similarly, there has been effort to understand “triple bottom line” outcomes for achieving environmental effectiveness of conservation initiatives while promoting equity in the participation and outcome of conservation efforts at least cost [23,24]. While these efforts are laudable, they have not had an explicit procedural lens and have focused on relatively narrow dimensions of success of individual conservation initiatives rather than broad and multiple social-ecological criteria found in the SDGs. These procedural problems are not trivial, as addressing the “3 pillars” of sustainable development (economic viability, social equity, and environmental sustainability) [25] in the wrong order may backfire. For example, cases exist where the establishment of protected areas without considering how social dynamics and local resource access would be changed as a result have led to conflict between conservation groups and communities and ultimately increased levels of poaching [26]. Thus, the uncertainty in procedural steps between environmental, social, and economic priorities towards an equitable and sustainable future has led to different and competing plans for implementing sustainable development in practice.

**Fig 1:**
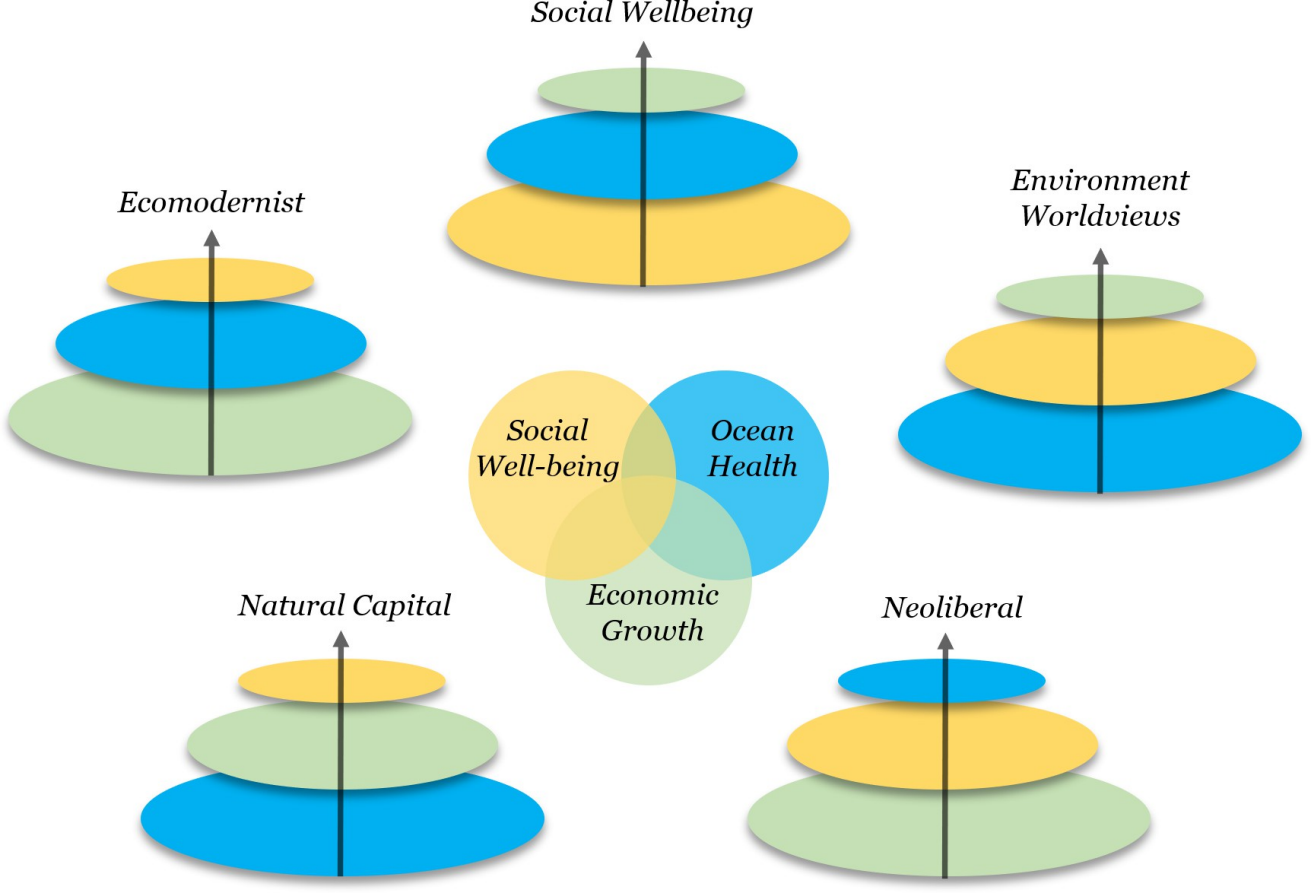
Five logic models for sustainable ocean development. Three dimensions of sustainable ocean development—social well-being, economic growth, and ocean health—and proposals for how to prioritize them according to 5 key development approaches and related ocean frameworks. The arrows represent the procedural dynamics of the logic models, and the pyramid-like structure of the assemblage of dimensions represent the logic that later dimensions depend on foundations of the earlier dimensions. Smaller and later dimensions in any logic model depend on the larger and more foundational dimensions that precede them. The names of 3 logic models mostly follow established terminology (Neoliberal, Natural Capital, Ecomodernist), and we chose to elect names for the final 2 with less of a history in the marine literature. Environment Worldviews represents a logic model that is primarily focused on natural resource conservation and management through leveraging the different worldviews of people around the world, and the Social Well-being model prioritizes redressing historical injustices and promoting diverse voices in development decisions. More explanation of each logic model is found in the text.

In this Essay, we compare 5 competing logic models of sustainable development for ocean and coastal systems and assess the ability of these models to address equity concerns in sustainable development, such as people’s inclusion in decisions that affect them, making decisions through fair and non-discriminatory policy, and people avoiding unjust burden of risks and impacts while enjoying their share of benefits. While the logic models we evaluate are all interpreted through the famous 3 pillar framework of sustainable development, the 5 distinct logic models consider the relationships and dependencies among these 3 pillars differently (Fig 1). In the context of our discussion about sustainable development policy for oceans, logic models are thus strategically informative. We first explore these models in the context of ocean development and marine conservation and address their potential contribution towards societal outcomes, then we discuss how global commitments to sustainable development post-2030 could be structured.

### Five logic models for sustainable ocean development

Although we focus our discussion on 5 idealized yet highly recognizable approaches to sustainable ocean development, we acknowledge that there are in fact 6 potential ways to link the 3 pillars, but only 5 have an existing theoretical and empirical literature associated with them.

The Neoliberal logic model sequentially prioritizes aggregate economic growth, with benefits sharing considered next (or assumed to lead to increased average incomes and well-being), leading to pro-environmental attitudes over time. Generally, this logic model follows the analogy that “a rising tide lifts all boats,” assuming that a growing economy benefits everyone. This logic model is largely represented in “free market” policy approaches that limit regulation or policy planning on resource development and allow economic growth to follow its own course. By contrast, the Ecomodernist logic model sequentially prioritizes economic growth through market-based and industry innovation focused on environmentally benign (or lower-impact) technologies, and society benefits through larger economies and cleaner environments. This logic model thinking posits that technological innovation that favors environmental protection and resource efficiencies will allow economic growth and environmental protection to go hand in hand, such that environmental issues are incorporated into markets [27–31].

The Natural Capital logic model sequentially prioritizes environmental protection and sustainable natural resource use in order to contribute to economic market and non-market values. Society then benefits through better connecting the natural environment to the economy. The logic follows the 1972 Limits to Growth thesis that the natural environment places physical constraints on the growth of the economy and also the subsequent argument at the heart of the Millennium Ecosystem Assessment: that human well-being depends on intact ecosystems [32,33]. This model also builds on the environment–conflict model of peace, whereby resource scarcity drives conflict and resource abundance sets the stage for peace [34]. This approach to sustainable development is also the best suited to acting on the notion of “strong sustainability,” whereby natural resources are thought to be non-substitutable and sustainability therefore means living only with renewable resources and only using them at a rate they can be replenished [35]. The Natural Capital logic model also highlights the infrastructural costs that can be avoided by “services” provided by intact ecosystems as well as the bequest values of having species not go extinct that may become important resources in the future.

The Environment Worldviews logic model sequentially prioritizes environmental protection and management, taking into account diverse values among diverse cultures. Aggregate economic benefits are increased as specific and local values (disaggregate values) are met. The perspective can be found within the field of ecological economics and one incarnation is the Nature’s Contributions to People, which features prominently in IPBES [36]. Although this model can be considered an offshoot of the concepts of ecosystem services and natural capital, it’s major point of departure from the Natural Capital model is to explicitly filter the benefits of natural resource conservation and management through the lens of diverse cultural perspectives.

Lastly, the Social Well-being logic model sequentially prioritizes local, cultural, and political inclusion in decision-making so people can address their own priorities and management of natural resources and environment. Aggregate economic growth is a reflection of local people’s well-being being met through recognition and dignity as well as outcomes of development. Placing social equity at the core of a sustainable ocean economy, with ocean health and economic growth as subsequent outcomes, has been argued to be essential for making the Blue Economy a truly transformative approach [37]. Although one final logic model exists in theory (in which society is prioritized above economic and then environmental concerns), we did not find evidence for it in existing approaches and initiatives. Given that societal-led approaches focus on disaggregated decision-making, it may be unlikely to address aggregate economic issues before environmental concerns, and this logic model may therefore not be operationalizable.

These 5 models have similarities and differences between them that manifest through their applications to ocean development. For example, the Natural Capital and Environment Worldviews models are informed by ecological economics and argue that social and economic dimensions are limited by biophysical constraints, thus positing environmental sustainability as more fundamental than the other 2 dimensions. The notion of biophysical limits of social and economic dimensions is found in some prominent development frameworks, notably the Planetary Boundaries framework and the various later frameworks that Planetary Boundaries influenced [38–41]. However, the Natural Capital and Environment Worldviews logic models make different assumptions of how equity is progressed (through active engagement in the Environment Worldviews model versus as people benefitting as a whole through increased benefits from natural ecosystems in the Natural Capital model). Further, the main difference between the Ecomodernist logic model and the Neoliberal logic model (both of which begin with a focus on economic growth) in the ocean context is that the former is much more directly focused on actively using environmentally friendly technologies in economic growth to decouple economic growth and environmental degradation, but the latter assumes that, once economic growth has led to a more developed and materially comfortable society, it will then be naturally interested in and able to promote ecological sustainability and conservation [27]. However, the technological focus of the Ecomodernist logic model and the financial focus of the Neoliberal logic model often do reinforce each other in practice through appeals to innovation and growth [8,12].

### Outcomes of logic models for ocean development on SDGs and equity

The 5 logic models reflect their assumed procedural frameworks to implement sustainable development, so their logic should be in service of the societal outcomes of the SDGs. In particular, the SDG focus on “no one left behind” places special emphasis on equity outcomes including the recognition of the concerns of culturally and politically marginalized people due to systemic issues including racism, gender discrimination, and guarded political power [10,42]. These and other issues manifest as the political exclusion of people in decision-making and political oppression against marginalized and vulnerable people. At national levels, the equity-based definition of sustainable development of the SDGs aims to promote small islands and least developed nations in regional and global decision-making and protect them from harms (including the trade-offs that come from the disproportionate role they have in conservation areas that limits development) while benefitting from development opportunities [43–45]. In this light, we provide a preliminary consideration of the ocean-related outcomes of interventions based on these logic models. In practice, there can be overlaps and gray areas between these models, some programs can try to employ multiple models simultaneously (which may compete), and some initiatives we indicate as operationalizing a certain logic model can adopt other logic models as they develop. For example, the Natural Capital Project, which bases its projects on ecosystem services, has adapted its approach over the years so that stakeholder engagement is used to define the environmental decisions and scope before deploying ecosystem service tools for calculations (following an Environment Worldviews model), whereas their approach used to be more focused on building tools to calculate changes in ecosystem services as initial stages (following a Natural Capital model) [46,47]. However, despite these overlaps and blending of different models, it remains important to reflect on the overall procedural perspective of initiatives to avoid trade-offs between outcomes for the 3 pillars.

### Neoliberal

This logic model largely follows the environmental Kuznets curve (EKC) logic. The EKC is a development–historical theory that argues that rising economic affluence will lead to social prerequisites needed to promote environmental sustainability [27]. The EKC posits that as societies grow their economies, there is usually an initial deterioration of the natural environment, but as economic development continues, the society’s relationship to the environment improves and environmental deterioration falls. On a graph of environmental impact over time, the EKC shows as an inverted “U” shape. EKC logic reasons that rising economic affluence will lead to social prerequisites needed to promote environmental sustainability. For example, growing the economy requires the expansion of markets, which promotes rational management of resources and investments in more efficient technologies [48,49]. Economic growth also portends a growing class of affluent, environmentally conscientious consumers who value sustainability in their purchases [50,51]. Moreover, economic growth’s early, dirty, phases of development eventually require clean up and improved management from increasingly eco-conscious civic and governance institutions [52,53]. We therefore emphasize that the Neoliberal Approach regards the EKC effect as a developmental process baked into the long-term logic of economic growth. An example of this logic model can be found in the ongoing negotiation around the use of marine genetic resources in the high seas. Extraction of marine genetics is promoted for aggregate economic gain, while discussion is focused on how benefits are to be shared after extraction rather than how and under what conditions such resources should be extracted [54].

However, evidence on the generalizability of an EKC relationship existing between economy and ecology is limited. A recent meta-analysis illustrates that an economic–environment decoupling trend occurs most frequently in certain atmospheric indicators, but its presence in land, ocean, and coastal-oriented indicators is sporadic [27]. Furthermore, the premises of the EKC have long been challenged on theoretic grounds. EKC processes in one or a few countries or industries, for example, do not necessarily suggest generalizable processes associated with all economic growth trajectories [55]. Furthermore, EKC studies should consider the rate and level at which decline occurs. Calculations of the EKC of fisheries footprint, for example, reveal more of a plateauing effect than an inverted U-shaped curve [56], and there is strong evidence suggesting that these footprints are merely exported to other locales rather than actually declining.

Scholars of development and global environmental justice also reason that the potential of a decoupling effect must be evaluated comparatively and relationally. Some studies illustrate a “trans-national tilt,” in which the environmental impact of economic growth increased absolutely in magnitude over time, but only in less-affluent countries [56,57]. Similar work illustrates how mechanisms of neoliberal development and growth in a modern, global economy foster inequitable trade dependence and enable affluent, powerful nations to displace their environmental impacts of consumption [58–60]. All in all, these patterns of development constitute what global environmental justice scholars refer to as unequal ecological exchange or the patterned, asymmetrical transference of biophysical wealth from poorer to richer countries across the global economy [61]. As such, the Neoliberal approach could foster positive outcomes in one nation, region, or industry but negative outcomes in others.

Characterizations of the EKC effect thus strongly correspond with the researcher’s theoretical orientation. As such, and in light of the growing urgency associated with promoting sustainable outcomes, economy-first plans are seeking economic growth trajectories that operate within ecological boundaries, an approach similar to that of Ecomodernism but weaker in terms of an explicit commitment to environmental restoration.

### Ecomodernist

In the context of ocean development, this logic model is reflected in current “blue growth” policies, such as the EU Blue Growth strategy that considers ocean sectors as new sources for environmentally sustainable growth that can produce economic surplus, jobs, and reduce poverty [62,63]. The key to achieving economic growth and well-being in this approach is the explicit use of ecologically friendly technologies and market incentives towards resource sustainability and conservation financing [64,65]. Such plans often characterize economic growth and ocean sector sustainability as mutually inclusive and, indeed, co-dependent. This logic contains some common sense appeal; after all, it is in the long-term economic interest to sustain underlying renewable natural resources, and in market economies the best way to effect change is through the market itself. Yet, a potential, unintended consequence of this approach is the relegation, and frequent omission, of social equity concerns from Blue Growth oriented developmental plans [8,37], as well as clear plans for how current ocean sectors will transition to envisioned ones [66].

Such omissions may stem from a tendency in economic thought to over-value the potential of more sustainable economic growth to promote positive outcomes without direct measures or to assume that increased aggregate benefits will be felt throughout a population. Ocean economy plans that emphasize business and economic growth are weakly associated with actionable calls and proposals to promote social equity [67]. Planners relying on the Blue Economic Growth approach tend to characterize the ocean as an emergent source of new value added potential, but often neglect to explain or propose clear pathways to achieving just and equitable outcomes [68]. In emphasizing emerging sectors, Blue Growth approaches therefore risk overlooking pathways—if not entire sectors, as in the EU’s initial exclusion of fisheries from its Blue Economy plans—that do not promise impressive and immediate extrinsic growth [69]. Indeed, the promotion of economic growth in already stressed environments is a key challenge for 21st Century Blue Growth.

### Natural capital

This logic model places environmental objectives in the foreground. This orientation thus differs from the Ecomodernist and Neoliberal approaches, which more explicitly prioritize the economy. However, there is much divergence on how to regulate ecological wealth and practically formulate/implement Natural Capital approaches [70–72]. Such debate is not surprising, given that scholarship on the subject has varied widely in its characterization of the price mechanism’s potential as a logically appropriate operationalization of ecological use value [73–76]. These approaches either (or simultaneously) argue that there are biophysical limits to economic growth or emphasize the benefits of natural systems to material well-being.

Natural capital initiatives often valorize marine ecosystems services in order to incentivize the creation of marine protected areas (MPAs). For example, the creation of a blue carbon commodity market has been posited as a mechanism to finance marine protection in ecologically vital coastal marine systems [77,78]. In doing so, conservation of large swaths of ocean territory could receive lucrative investment to ameliorate economic pressure to undergo “business as usual” development. However, evidence of sustained market investment and profit-making from such measures is limited, and success of program development can hinge on accommodating alternative resource uses for local people and prioritizing social benefits over profit [79–81]. Thus, despite common claims about the amount of untapped economic value of marine ecosystem services, the profit potential of natural capital valuation faces notable obstacles, and only very few communities have truly established sustainable business models from ecosystem services [8].

Ironically, recent developments in ecosystem services theory reveal how natural capital approaches may overestimate the benefits from protected nature. Since ecosystem services are the services that natural ecosystems render to people, the value of ecosystems are only realized when people can access them [82]. Many ecosystem services studies only consider the production of services in their estimates, therefore overestimating their value. For example, MPAs risk denying local people access to marine resources that sustain material and cultural reproduction [20]. Disrupting users’ access to resources often corresponds with ocean-grabbing or a form of imposed marine dispossession that enables capital accumulation at the political, economic, and cultural expense of small-scale, local users [83,84]. States, firms, and large NGOs often lead the creation of MPAs in a top-down fashion, fail to incorporate local concerns into the initial planning and, relatedly, neglect to grant artisanal fishers rights to access [26,84,85].

One of the most frequently employed interventions employing the Natural Capital logic model in the ocean realm is the creation of a catch share or transferable quota systems (ITQ). ITQs are intended to address issues of long-term resource sustainability by ensuring that catches of fish stocks are limited by tying quota to a total allowable catch (TAC) based on biological limits of population sustainability, with markets set up for trading individual quotas. In addressing long-term resource sustainability, they may address intergenerational equity concerns. However, ITQs can exacerbate social injustice and inequity in fishery systems by emphasizing historic and contemporary inequities [86]. Catch share programs often consider fisheries, and those who access them, as homogenous groups comprising like-minded, rational actors of equal socioeconomic standing. Environmental anthropologists, for example, have long contended that ITQs over-simplify social and community dynamics, and incorrectly assume that privatization of commonly held resources intrinsically leads to better management [87,88]. The share of allowed catch (based on the TAC) is divided between prior users, favoring already established fishers and well-capitalized firms [89]. Smaller scale fishers, or those seeking to initiate access to fishery resources, can become unable to afford entry. Often, this results in the selling or leasing of fishery rights to larger, more affluent, and non-local firms [89,90]. Thus, critics of ITQs illustrate how quota-share systems can inequitably extract wealth from fishing communities, threaten the economic viability of small scale fishers, and jeopardize access customs utilized by coastal Indigenous Peoples [91–93].

### Environment worldviews

Similar to the Natural Capital model, the Environment Worldviews model is focused on environmental protection and sustainability; however, instead of presupposing the kinds of interventions needed through economic analysis, this model works with local and regional decision-makers to understand their priorities for environmental management before working through potential interventions and ways forward. The Nature’s Contributions to People (NCP) framework, advocated by IPBES, seeks to understand how people benefit from natural systems through the explicit consideration of different cultural lenses, to inform development policy, partly through an explicit lens of “relational values” that track the values that people have in relationship with the natural world and each other [36]. This framework shows promise in advancing equity considerations to include multiple worldviews and local issues, especially since the 3 previously discussed logic models largely neglect the existence or importance of worldviews and broad participation in decision-making. While there have not been many applications of the NCP approach (given how recent it is), there exist some early criticisms of how well it captures the diversity of different cultural lenses, given that the framework is largely constructed by researchers from the west and global North. For example, some studies suggest that the NCP framework can reaffirm western values and notions of human–nature relationships in practice and are not easily inclusive of other values [94,95].

The Environment Worldviews model has also been applied in specific environment and development planning projects around the world. The Natural Capital Project has been working with communities around the world on projects, aiming to illuminate the benefits people receive from natural systems, and working through continuous engagement with communities to create planning models to aid in development decision processes [96]. The tools developed in this process have led to climate and environmental planning in Canada, the United States of America, Costa Rica, Kenya, and other locations [97–99]. The application of the Environmental Worldviews model through collaborative environmental planning seems to be able to deliver on-the-ground sustainable development planning, including community involvement, institutional support, and developmental outcomes, so long as the project is predetermined to focus on environmental goals and environmental protection.

### Social well-being

As shown in Fig 1, the Social Well-being logic model is unique as the only model we identify where social considerations are not preceded by environmental or economic ones. In the ocean realm, there is a large body of evidence at local scales highlighting the fundamental benefits of this approach for social cohesion and well-being, often including but potentially separate from environmental or economic outcomes. This includes movements related to Indigenous resurgence in coastal areas [19], fishing and tourism cooperatives and other collectivist arrangements [18], and the recognition and incorporation of traditional resource and coastal management approaches and ways of knowing [100,101]. While this model has not been implemented at larger scales, the focus on social equity as an intrinsic goal beyond environmental sustainability or economic viability has been increasingly recognized, including in the FAO Voluntary Guidelines for Small Scale Fisheries [102], the Monterey Framework for Socially Responsible Seafood [103], and key reports of the High-Level Panel on a Sustainable Blue Economy [5]. As a bottom-up approach, it may also be the only logic model capable of fully delivering on the SDG target to promote the social and political inclusion of everyone (SDG 10.2), and specific interventions focused first on addressing social policy may be best suited on the SDG target focused on reducing discriminatory processes (SDG 10.3). Indeed, recent global analysis suggests that functioning institutions, legal protections for human rights, reduced corruption, and economic equity are larger barriers for achieving equitable and sustainable blue economy visions than natural resource availability [37]. Furthermore, although resource availability for all ocean sectors is not equally distributed across the world, resource availability for some ocean economies is typically not limiting. However, differences in regional capacity to develop sustainable and equitable economies is often due to institutional and social equity differences [37]. Similarly, global analyses of MPAs show that performance of protected areas (towards ecological conservation) is often limited by social and governance factors (such as staff capacity, budget, and inclusive decision-making including communities) more than by the biophysical conditions of the site being protected [104].

At local scales, this logic model is well represented by examples from various development initiatives in which Indigenous communities around the world seeking and asserting sovereignty have leadership over their own resource use and developments to decide on development [19,105,106]. Current movements towards Indigenous governance through national and international efforts (such as those supported through the UN’s Declaration on the Rights of Indigenous Peoples) support the reestablishment and assertion of Indigenous sovereignty [107]. A study looking at Indigenous-led UNEP Equator prize winners outlines various strategies Indigenous groups around the world use towards marine management, through negotiating or otherwise asserting their rights with governments [19]. While the development of Indigenous-led impact assessment is often constrained, it does allow a mechanism for front-ending social equity concerns regarding large-scale resource development [108]. In opposition with the Environment Worldviews model, which can view Indigenous people as inherent environmental conservationists and advocating for Indigenous rights as a secondary goal towards the primary goal of environmental protection, the Social Equity lens recognizes that Indigenous communities can be pro-industrial development in order for greater employment and social stability in the face of historic dispossession of autonomy and rights [19]. The explicit “bottom up” structure of the Social Well-being model, led by diverse communities rather than top-down decisions and specific goals, means that outcomes beyond social inclusion and equity can be less predictable.

## Conclusions

While the broad concept of sustainable development has widespread appeal, there are competing models to approach the goals of sustainable development. The different logic models we have identified developed over time in response to a changing and broadening definition of “sustainable development,” though all purport to address issues of social equity—sustainable development’s ultimate objective. Interrogating the procedural structure of logic models in these different approaches is imperative to plan for effective interventions towards equitable sustainable development. As ocean and coastal researchers, we have stressed the importance of marine systems, but acknowledge the importance of other systems towards sustainable development [10], and welcome other system-focused reviews of sustainable development models to further understand how to achieve a more sustainable and equitable world. The complex dynamics of sustainable development are path dependent, and well-intentioned initiatives can fail or backfire, but a major lingering problem is that most models lack evidence of operational efficacy or are driven by assumptions and limited theory [11,12].

The 5 logic models of ocean sustainable development we have discussed emphasize different pathways and dynamics towards equity. While economic-based approaches, such as the Neoliberal and Ecomodernist logic models, emphasize economic growth as necessary for everyone to benefit, the 2 differ in how planned this growth should be. Neoliberal logic models assume a historic pro-environmental trajectory that is an inevitable product of economic growth, while Ecomodernist models specifically plan for environmentally benign (or low impact) technologies. Overall, there is noticeable criticism of aggregate-economic-based development policy and intervention, as these policies are arguably the reason that sustainable development is now needed [53]. Immense economic growth has led to many of the social inequities and environmental challenges we face today [52,53]. However, in light of growing awareness of human impacts on the environment globally from economic activity, the Ecomodernist approach is one of several to highlight the need for specific attention on limiting environmental impact (though it still emphasizes the need for economic growth).

The Natural Capital and Environment Worldviews logic models, in contrast, make environmental protection the immediate focus. Following a focus of sustainable development focused largely on the maintainability of natural systems in order to guarantee equity of intergenerational resource opportunities, these models follow ecological economic assumptions about natural limits to economic growth [109]. Such environmental-limits thinking can often fall prey to first-principles logic that neglects feedbacks and higher-order dynamics [11]. For example, if the idea that the social sphere is merely a subset of the environmental is taken too seriously, it can lead to thinking that social dynamics are limited by environmental capacity, and therefore, social considerations are secondary concerns [11]. However, there is considerable evidence that environmental programs fail precisely because of a lack of social and economic infrastructure or support [12,17,26,37,104]. Furthermore, the shocks of Coronavirus Disease 2019 (COVID-19) and the associated inequitable economic and public health consequences have—in a single year—reduced or reversed gains made against the SDGs since 2015 (most notably in reducing poverty and hunger and reducing global inequalities) and led to some reduced environmental protections to encourage economic growth [110,111]. We are unconvinced that greater environmental protections would have limited the unequally distributed impacts of COVID-19 as much as stronger institutions and public health protections for people would have.

While models with active environmental protection as an immediate focus may be more effective for addressing environmental degradation, they have been criticized for interpreting sustainable development as merely (environmental) harm reduction, which is uninspiring, unengaging, and denies human agency towards solving problems (even problems humans caused) [13,112]. Those in the development community point out how undesirable the world would be if development followed models of environmental limits: in particular because halting global development would stagnate the economic prospects of least developed countries (LDCs), Small Island Developing States (SIDS), and marginalized groups within all nations, enforcing global inequities and favoring those groups and nations that had a head start in depleting their own resources and releasing emissions [40]. Unsurprisingly, these models have had very limited uptake in actual development policy and will likely continue to be rejected [40]. Moreover, where these models focus on increasing aggregate benefits or avoiding aggregate losses (more so in the Natural Capital logic model), they can enhance inequities [86,92]. By contrast, the Environment Worldviews logic model shows some successes in promoting community well-being and equity through increasing local decision-making; however, the model has not been investigated in cases where people’s concerns are not primarily environmental or may even be against immediate environmental goals. Is it a model with limited applicability, or a model that forces all problems through a limited purview, or something else? Given that it shows some evidence of potential success in limited cases and is a relatively new approach, more research is needed to examine this logic model and its effectiveness to promote equity broadly.

The Social Well-being model is the only logic model we investigated that is purely bottom-up, starting with community concerns (and not presupposing immediate economic or environmental priorities). In this way, it can bypass the kind of “first-principles” thinking that sometimes dominates environment-based models and tries to emphasize not just the “maintainability” of sustainable systems but also put focus on desirability as well [11,109]. Being the only bottom-up approach, this model may be the only model capable of delivering on certain SDG targets (specifically targets focused on equitable process, such as SDG 10.2 and 10.3). Outside of the limited marine examples that exist, the Social Well-being model is also reflected in the literature on “regenerative sustainability” that has largely developed in the context of the built environment [113]. Regenerative sustainability is a participative framework that emphasizes democratic and equitable process over eventual outcomes, directly responding to major shortcomings to sustainable development practices over time: namely that the aspirations and societal goals of sustainable development (especially at global levels) are vague, foster delusions around what is achievable, provide no guidelines for actions, and often “greenwash” development [13,112,113]. However, despite the promises, this model is the least explored in a marine context and, as a bottom-up approach focused on community needs, faces significant uncertainties in scaling up to global challenges.

Different logic models have different disciplinary origins and are legacies of distinct historical thought. Given the promise of the Social Well-being logic model and the (perhaps more limited) potentials of the Environment Worldviews logic model towards equity, sustainable development initiatives may be more successful if they took on these logic models. Adopting new or different logic models will require active experimentation to adopt into new contexts, which will face resistance within professional cultures and will need to overcome institutional inertia. For example, a perspective using the Environment Worldviews logic model has already faced resistance from those who work within the Natural Capital perspective [114]. Such considerations of how to enact change or adopt new perspectives are worthy of a unique and different review than this one. We hope that the interdisciplinary and transdisciplinary exercise of this Essay at least broaden awareness of alternative approaches that can foster some of the experimentation needed to lead sustainable development initiatives to better deliver on equity promises.

Despite repeat global initiatives to promote sustainable development, the history of sustainable development has largely been a history of failure to create an equitable planet [11–13]. We contend that part of this failure is due to a reluctance to examine the logic models that policies and interventions are based on. Perhaps one central conclusion of our review of different logic models is that models that front-end social equity procedurally (the Environmental Worldviews and the Social Well-being logic models) are best positioned to address equity and not backfire. Models that focus on aggregate outcomes and assume equity outcomes will manifest later can easily reinforce inequities. However, throughout the history of sustainable development, social equity has been procedurally neglected [115]. Even models emphasizing intergenerational equity can fail if they do not address contemporary social equity concerns and inclusion. That is, it may be difficult, if not impossible, to achieve intergenerational equity without addressing contemporary inequities and historic injustices. The SDGs are set to expire in 2030, but to protect the gains made towards them, reverse the declines away from them, and to improve beyond 2030 against the goal to leave no one behind, we will need to employ the right procedural strategies and understand how best to operationalize them.

## Abbreviations:

COVID-19: Coronavirus Disease 2019
EKC: environmental Kuznets curve
GDP: gross domestic product
IPBES: Intergovernmental Science-Policy Platform on Biodiversity and Ecosystem Services
LDC: least developed country
MPA: marine protected area
NCP: Nature’s Contributions to People
SDG: Sustainable Development Goal
SIDS: small island developing states
TAC: total allowable catch
